# Early treatment-seeking behaviour for malaria in febrile patients in northwest Ethiopia

**DOI:** 10.1186/s12936-018-2556-2

**Published:** 2018-11-03

**Authors:** Baymot Workineh, Fantahun Ayenew Mekonnen

**Affiliations:** 1 Field Epidemiology Program, College of Medicine and Health Sciences, University of Gondar, Gondar, Ethiopia; 2 Department of Epidemiology and Biostatistics, College of Medicine and Health Sciences, University of Gondar, Gondar, Ethiopia

**Keywords:** Malaria, Early treatment-seeking behaviour, Associated factor, Ethiopia

## Abstract

**Background:**

As malaria is among the leading public health problems globally, early diagnosis and treatment of cases is one of the key interventions for its control and elimination. Nevertheless, little is known about early treatment-seeking behaviour for malaria of people in Ethiopia. This study was conducted to investigate early treatment-seeking behaviour and associated factors among febrile patients in Dera district, one of the malaria hotspot districts in Ethiopia.

**Methods:**

An institution-based, cross-sectional study was conducted among malaria-suspected febrile patients in Dera district, Amhara Regional State, Ethiopia from September to December 2017. The study used the lottery method to select sample health facilities, and participant allocation to facilities was done in proportion to client flow to the respective health facilities. Data were collected by interview. Thus, binary logistic regression model was fitted to the data. Crude and adjusted odds ratios with the respective confidence intervals and p-values were computed. An explanatory variable with a p-value ≤ 0.05 was considered statistically significant. SPSS version 20 was used for the analysis.

**Results:**

A total of 680 respondents completed the study with a response rate of 96.6%. The study revealed that 356 (52.4%) participants sought treatment within 24 h of fever onset, and patients who: knew the advantage of sleeping under nets [AOR 95% CI 2.8 (1.70–4.60)]; knew mosquito breeding sites [AOR 95% CI 1.9 (1.10–3.30)]; had good, overall knowledge about malaria [AOR 95% CI 2.7 (1.56–4.76)]; had previous history of malaria [AOR 95% CI 3.26 (1.64–6.49)]; were at a distance of < 6 km from a health centre [AOR 95% CI 2.5 (1.72–3.60)]; and, had family size < 5 [AOR 95% CI 2.1 (1.43–3.20)], were more likely to seek treatment within 24 hof fever onset.

**Conclusion:**

A low proportion of malaria-suspected patients sought treatment within 24 h of fever onset compared to the national target. Awareness about the advantage of sleeping under nets, knowledge about mosquito breeding sites and malaria itself, previous history of malaria, distance from the health centres, and family size were found to be predictors of early treatment-seeking behaviour for malaria. Strengthening strategies tailored to increasing awareness for communities about malaria prevention methods and early treatment-seeking behaviour is essential.

## Background

Malaria is a leading public health problem, reported to have caused 212 million new cases and 429,000 deaths globally in 2015; 90% of cases and 92% of deaths were in Africa [1]. In Ethiopia, malaria has been reported to be the consistently leading cause of morbidity and mortality. In 2014/2015, 2,174,707 cases and 662 deaths were reported (Amhara Regional State recorded 610,486 (28%) cases). Currently, 75% of the land mass and 60% of the population of Ethiopia are considered to be at risk of malaria [2].

Malaria is an epidemic prone but preventable disease. However, efficient malaria elimination and achieving a near-zero malaria death rate requires an integrated approach, including prevention (vector control) and prompt treatment with effective anti-malarial agents [3]. Early treatment seeking is believed to be critical behaviour that is helpful for the success and sustainability of malaria control efforts [4]. However, the literature has documented very slow progress in this perspective, unlike the achievements of vector control, due to a number of reasons. Some of the reasons are healthcare accessibility, knowledge and attitude towards malaria, and socio-economic characteristics [5–9].

A range of malaria prevention and control strategies have been designed and implemented in Ethiopia, and significant progress has been made in reduction of cases and deaths nationally [10]. However, the achievement has not been only as satisfactory as was expected, but is highly variable across regions and varying geographic areas [2]. In the current study area (Dera district), Amhara Regional State, the incidence of malaria is persistently high and reported to be the leading cause of morbidity. As a result, it has been distinguished as a hotspot of malaria in Ethiopia. In spite of this, data relating to timely treatment-seeking behaviour for malaria is limited in the country in general. No study has been conducted in the current study area although it is known to be a hotspot area for malaria. Therefore, this study was conducted to assess early treatment-seeking behaviour and associated factors among malaria-suspected febrile patients in Dera, northwest Ethiopia.

## Methods

### Study design and setting

An institution-based, cross-sectional study was conducted among malaria-suspected febrile patients visiting health facilities in Dera district, northwest Ethiopia, from September to December, 2017. The district is located 611 km from the capital of Ethiopia, Addis Ababa, and 47 km from Amhara Regional State capital, Bahir Dar. The total area of the district is about 1474 sq km with a total population of 284,701, out of which 261,231 are rural dwellers. The district has 29 rural and 3 urban *kebeles* (the smallest administrative units in the hierarchy of government administration). All of the *kebeles* are characterized as malarious. There are 47 governmental healthcare facilities (11 health centres and 36 health posts), 12 private clinics of which 2 are medium (a health-care facility whose services are restricted to providing outpatient services, while it must have a laboratory and a drug-dispensing unit, and should be staffed with health professionals at least of Bachelor-level qualification) clinics, and 10 low-level clinics in the district.

### Source and study populations

All patients who visited the health centres of Dera district with fever as a chief complaint, with or without other manifestations, during the current Ethiopian fiscal year (July, 2017 to June 2018) were the source population, while patients with the same chief complaint and who visited the same health centres in Dera district during the data collection period (September to December 2017) were the study population. Malaria-suspected febrile patients who had lived in the district for less than 1 year or those who were incapable of responding due to serious illness were excluded from the study.

### Sample size determination

Single and double population proportion formulae were utilized to calculate the sample size for the first (prevalence of early treatment-seeking behaviour) and the second (factors associated with early treatment seeking) objectives, respectively. Of the three factors (attitude to malaria, perceived susceptibility to malaria, and knowledge of the advantages of bed nets for the prevention of mosquito bite) [11] considered, perceived susceptibility to malaria yielded the largest sample size. The sample sizes obtained from the two formulae were compared with each other. The double population proportion formula (perceived susceptibility for malaria) resulted in a larger sample size (704) compared to the single population proportion formula (363). Epi info 7 was used for the sample size calculations.

### Sampling techniques

Initially, 4 health centres were selected out of 11, using the lottery method, after obtaining the names of all health centres from Dera District Health Office. The calculated 704 sample was distributed to 4 randomly selected health centres. The sub-sample size assigned to each health centre was determined based on the number of patients the health centre had received during last year’s similar month of the current study period (September to December, 2016). The 4 health centres with their sub-sample were Anmbesame (139), Hamusit (110), Arib Gebiya (253), and Gelawudiwos (202). The sample population was composed of febrile patients with fever as the main complaint receiving care at the selected health centres. All malaria-suspected febrile patients who visited the selected health facilities during the study period were included in the study.

### Data collection methods and instruments

A structured questionnaire was prepared, originally in English and then translated to the local language (Amharic). Data collectors received 2 days training. The questionnaire was pre-tested on patients with fever as chief complaint but who had visited one of the health centres in Dera district other than the 4 randomly selected health centres. Data were collected on treatment-seeking behaviour: as early if a patient came to the study health centre within 24 h of fever onset, or as late if the patient came after 24 h of fever onset. Data were also collected on participants’ knowledge of and attitude to malaria and perceived susceptibility to malaria. The definitions of these key variables were adapted from World Health Organization guidelines [3] and previous similar studies. Patients that were 15 and above years old were directly interviewed, while the parents/care takers were interviewed for patients of under 15 years old.

### Data management and analysis

Data were checked for completeness and consistency, then coded and entered into epi info 7 and exported to SPSS version 20 for analysis. Descriptive analyses were carried out to see the frequency distributions of different characteristics of respondents. Bivariable and multivariable binary logistic regression analyses were performed to estimate the effects of each explanatory variable on the dependent variable. Odds ratio to measure the associations between the dependent and independent variables, and 95% confidence interval to see the stability of the odds ratio were computed by the models. A variable with p-value of 0.2 or below in the tests of significance in the evaluation of the association of the independent variables with early treatment-seeking behaviour for malaria in the bivariable analysis were exported to the multivariable regression model. An explanatory variable with a p-value < 0.05 in the final model (multivariable regression) was considered statistically significant. The final model was assessed for its fitness to the data by Hosmer and Lemeshow’s goodness of fit test. A test of interaction was also performed.

### Ethical issues

Ethical approval was obtained from the Institution Review Board of the University of Gondar, and a letter of cooperation was written to the Amhara Region Health Bureau. Letters of permission were obtained from the Regional Health Bureau, South Gondar Zone Health Department and Dera District Health Office. Letters of cooperation were written to selected health facilities by the Dera District Health Office. Finally, informed consent from study participants of 18 and above years old and ascent from below 18 years old participants was obtained. Respondents’ participation in the study was completely voluntary. They were informed that they could refuse participation in the study. They were told they could interrupt the interview at any time. The information collected was kept strictly confidential.

## Results

### Socio-demographic characteristics

A total of 680 participants completed the study, with a response rate of 96.5%. The median age of respondents was 34 years, the youngest 3 years old and the eldest 84 years old. Two-thirds, 421 (61.9%), of participants were male. Out of the total 680 respondents, 610 (89.7%) were rural dwellers. Two-fifths, 272 (40%), were able to read and write. More than half, 370 (54.4%), were farmers, followed by daily labourers, 88 (12.9%). More than two-fifths, 298 (44%), had an average monthly income of < $23. Two-thirds of participants, 408 (60%), were married, and 350 (52%) had fewer than 5 family members. Three-hundred and seventy-one (54.6%) participants came from fewer than 6 km from a nearby health centre (Table 1).

**Table 1:** Socio-demographic characteristics of febrile patients, northwest Ethiopia, September to December, 2017

Variables	Frequency (%)
Age (in years)	
< 5	27 (4.0)
5–14	36 (5.3)
≥ 15	617 (90.7)
Gender	
Female	259 (38.1)
Male	421 (61.9)
Residency	
Rural	610 (89.7)
Urban	70 (10.3)
Educational status	
Unable to read and write	207 (30.4)
Able to read and write	272 (40.0)
Not applicable	27 (4.0)
Primary	119 (17.5)
Secondary and above	55 (8.1)
Occupation	
Daily labourer	88 (12.9)
Farmer	370 (54.4)
Government employee	18 (2.7)
Merchant	63 (9.3)
Housewife	34 (5.0)
Not applicable	31 (4.5)
Student	76 (11.2)
Average monthly income	
< $24.9	298 (44.0)
$25–49.9	273 (40.0)
≥ $50	109 (16.0)
Marital status	
Single	139 (20.4)
Married	408 (60.0)
Divorced	31 (4.6)
Widowed	28 (4.1)
Not applicable	74 (10.9)
Family size	
≥ 5	330 (48.5)
< 5	350 (51.5)
Distance of health facility (km)	
≥ 6	309 (45.4)
< 6	371 (54.6)

### Knowledge about malaria

**Fig. 1:**
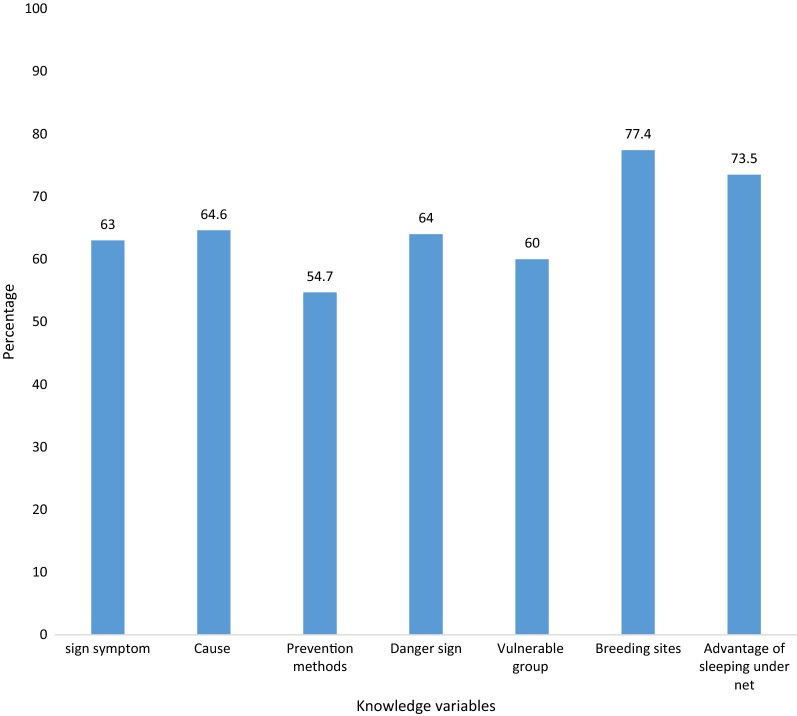
Knowledge of febrile patients about malaria, northwest Ethiopia from Sept to Dec, 2017

Most, 674 (99%), of participants knew fever is the main symptom of malaria, followed by 608 (89.4%) who reported it as headaches. Over two-thirds, 439 (64.6%), recognized that malaria was transmitted by mosquitoes. The majority, 630 (92.6%), reported seizure/convulsion as a danger sign of malaria followed by 510 (75%) who reported this as high-grade fever. Over two-thirds, 476 (70%), mentioned pregnant women as the most vulnerable group, followed by 432 (63.5%) who said that children were highly susceptible (Table 2). Overall, 410 (60.3%), had good knowledge about malaria, of whom the majority (77.4%) knew about mosquito breeding sites, followed by 73.5% who recognized the advantages of sleeping under bed nets (Fig. 1).

**Table 2:** Knowledge of febrile patients about malaria, northwest Ethiopia, September to December, 2017

Knowledge characteristics	Frequency (%)
Knowledge of malaria symptoms	
Fever	674 (99.0)
Chills	264 (38.8)
Headache	608 (89.4)
Nausea/vomiting	170 (25.0)
Loss of appetite	387 (56.9)
Joint pain	263 (38.7)
Feeling weakness	82 (12.0)
Knowledge of malaria causes	
Mosquito bite	439 (64.6)
Eating immature sugarcane	158 (23.2)
Cold/changing weather	43 (6.3)
Drinking dirty water	33 (4.9)
Hunger (empty stomach)	5 (0.7)
Getting soaked with rain	2 (0.3)
Knowledge of malaria danger signs	
Seizure/convulsion	630 (92.6)
Any fever	177 (26.0)
High fever	510 (75.0)
Unable to eat	468 (68.8)
Vomiting	210 (30.9)
Chills/shivering	320 (47.0)
Do not know	5 (0.7)
Knowledge of vulnerable groups	
Children	432 (63.5)
Pregnant	476 (70.0)
Any person	178 (26.2)
Elderly	15 (2.2)
Knowledge of advantage of sleeping under net	
Knew advantage	500 (73.5)
Do not know advantage	180 (26.5)
Knowledge of mosquito breeding sites	
Stagnant water	538 (79.1)
Waste/polluted water	425 (62.5)
Broken glass plastics	225 (33.0)
Knowledge of prevention of malaria	
Sleep under a mosquito net	351 (51.6)
Avoid mosquito bites	191 (28.0)
Environmental control	578 (85.0)
Take preventive medication	49 (7.2)
Spray house with insecticide	584 (85.9)
Do not know	1 (0.2)

### Attitude about malaria, malaria severity and susceptibility perception

Concerning malaria severity, 393 (57.8%) of participants perceived malaria as a severe disease. More than half, 376 (55.2%), perceived that they were susceptible to malaria. Fifty-seven percent (390) had favourable attitude to treatment for malaria (Table 3).

**Table 3:** Level of malaria severity and susceptibility perception of febrile patients, northwest Ethiopia, September to December, 2017

Variables	Strongly disagree # (%)	Disagree # (%)	Neutral # (%)	Agree # (%)	Strongly agree # (%)
Perceived severity (Cronbach’s α = 0.93)					
Malaria is serious disease	24 (4.0)	201 (29.0)	146 (22.0)	275 (40)	34 (5.0)
Worried suffering from malaria	32 (4.7)	225 (33.1)	63 (9.3)	306 (45.0)	54 (7.9)
Complications of malaria are dangerous	11 (1.6)	164 (24.1)	115 (16.9)	282 (41.5)	108 (15.9)
Risk of death from malaria is higher in children and pregnant women	20 (3.0)	183 (27.0)	87 (13.0)	342 (50.0)	48 (7.0)
Severity of malaria is high among individual that do not seek treatment in time	16 (2.4)	208 (30.6)	85 (12.5)	293 (43.1)	78 (11.5)
Perceived susceptibility (Cronbach’s α = 0.95)					
Malaria is prevalent in your area	34 (5.0)	189 (27.8)	105 (15.4)	234 (34.4)	118 (17.4)
You are vulnerable to malaria	22 (3.0)	218 (32.0)	90 (13.0)	217 (32.0)	133 (19.6)
Fever could be due to malaria	33 (4.9)	180 (26.5)	82 (0.1)	277 (40.8)	108 (15.9)
Malaria can cause anemia	31 (5.0)	211 (31.0)	105 (15.0)	237 (35.0)	96 (14.0)
Children and pregnant women mostly affected by malaria	16 (2.35)	205 (30.2)	99 (14.6)	263 (38.7)	97 (14.3)
Attitude variables (Cronbach’s α = 0.93)					
Visiting HF within 24 h is better when you feel fever	6 (1)	164 (24)	108 (16)	280 (41)	122 (18)
Getting drug out of health professional prescription is not good for malaria	29 (4)	178 (26)	107 (16)	273 (40)	93 (14)
Visiting HF is better than home remedies?	3 (0.44)	204 (30)	108 (15.9)	352 (51.8)	13 (1.9)
Malaria drug is affordable at HF?	31 (4.6)	282 (41.5)	13 (1.9)	320 (47)	34 (5)
Taking malaria treatment from HF is better than traditional healer	8 (1.18)	131 (19.3)	162 (23.8)	319 (47)	60 (8.8)

### Treatment-seeking behaviour for malaria

Of the total 680 respondents, 356 (52.4%) sought treatment within 24 h of fever onset. Out of the 680 respondents, 79 (11.6%) sought some sort of treatment prior to coming to the study health centres; 56 (70.9%) sought home remedies, while the rest reported visiting spiritual or traditional healers (Table 4).

**Table 4:** Treatment-seeking behaviour among febrile patients, northwest Ethiopia, September to December, 2017

Variable	Frequency (%)
Treatment seeking	
Early	356 (52.4)
Late	324 (47.6)
Treatment attempted at home	
Home remedies	56 (70.9)
Traditional healer	23 (29.1)

### Factors associated with early treatment-seeking behaviour for malaria

The result of the multivariable logistic regression analysis showed that 6 explanatory variables, such as knowledge of the advantage of sleeping under bed nets, knowledge of mosquito breeding sites, overall knowledge about malaria, previous history of malaria illness, residence distance from the health centre, and family size were independently associated with early treatment-seeking behaviour for malaria among malaria-suspected febrile patients. Patients who knew the advantages of sleeping under bed nets and about mosquito breeding sites were 2.8 [AOR 95% CI 2.8 (1.7–4.6)], and 1.9 [AOR 95% CI 1.9 (1.1–3.3)] times more likely to seek treatment early compared to those who did not know the advantages of sleeping under bed nets or about mosquito breeding sites, respectively. Similarly, patients who had good overall knowledge were 2.7 times more likely to seek treatment early compared to those less knowledgeable [AOR 95% CI 2.7 (1.56–4.76)]. Patients who reported previous history of malaria were 3.3 times more likely to seek treatment early than who did not report previous history, 3.3 [AOR 95% CI 3.3 (1.64–6.49)]. In addition, patients who were within 6 km of a health centre and had fewer than 5 family members were 2.5 [AOR 95% CI 2.5 (1.72–3.6)] and 2 [AOR 95% CI 2.14 (1.43–3.2)] times more likely to seek treatment earlier than patients who were more than 6 km from a health centre and who had more than 5 family members, respectively (Table 5).

**Table 5:** Predictors of early treatment-seeking behaviour among febrile patients, northwest Ethiopia, September to December, 2017

Factors	Treatment-seeking			
	Early (%)	Late (%)	COR (95% CI)	AOR (95% CI)
Knowledge of advantage of sleeping under bed net				
Yes	316 (88.8)	184 (56.8)	6.0 (4.00–8.90)	2.8 (1.70–4.60)
No	40 (11.2)	140 (43.2)	1	1
Knowledge of mosquito breeding site				
Yes	319 (89.6)	207 (63.9)	4.9 (3.20–7.30)	1.9 (1.10–3.30)
No	37 (10.4)	117 (36.1)	1	1
Overall knowledge of malaria				
Good	71 (20.0)	199 (61.4)	6.4 (4.50–9.10)	2.7 (1.56–4.76)
Poor	28 5(80.0)	125 (38.6)	1	1
Previous malaria history				
Yes	262 (73.6)	98 (30.2)	6.4 (4.60–8.98)	3.3 (1.64–6.49)
No	94 (26.4)	226 (69.8)	1	1
Family size				
< 5	240 (67.4)	110 (34.0)	4.0 (2.93–5.54)	2.1 (1.43–3.20)
≥ 5	116 (32.6)	214 (66.0)	1	1
Residence distance (km)				
< 6	242 (68.0)	129 (39.8)	3.2 (2.34–4.40)	2.5 (1.72–3.60)
≥ 6	114 (32.0)	195 (60.2)	1	1

## Discussion

This study revealed that 52.4% (95% CI 50.5–54.3%) of participants sought treatment within 24 h of fever onset. Participants with < 5 family members, living < 6 km from a health centre, had previous malaria history, knew the advantages of sleeping under bed nets, knew about mosquito breeding sites, and had good overall knowledge about malaria were more likely to seek treatment within 24 h of fever onset.

The level of early treatment-seeking behaviour estimated by this study is nearly half lower compared to Ethiopia’s national target of diagnosing 100% suspected malaria cases using rapid diagnostic tests (RDTs) and/or microscopy within 24 h of fever onset [10]. This difference may be due to the fact that little emphasis was given to education and communication compared to vector control activities synergized by campaigns [2, 10]. However, the result of the study is higher than those of similar studies conducted in different parts of Ethiopia, for example, Bale zone (12.2%) [11], Adami Tulu district (13%) [12]. The better early treatment-seeking behaviour in this study may be due to more access to health facilities and the time of the study [13]. The current study result is in line with a study report in Guinea that 46.7% of children sought treatment within 24 h of onset of symptom [5].

Concerning factors associated with early treatment-seeking behaviour for malaria of febrile patients, knowledge of advantage of sleeping under bed nets was found to significantly increase the likelihood of early treatment-seeking behaviour for malaria compared to not having the knowledge. This is similar to the result of a study in Bale, southeast Ethiopia, which revealed knowledge of mosquito nets as a means of mosquito bite prevention was strongly associated with early treatment-seeking behaviour [11]. In this study, participants who had knowledge about mosquito breeding sites sought treatment more than participants who did not. Similarly, patients who had good overall knowledge were more likely to seek treatment within 24 h of fever onset than patients who had poor knowledge. This is in line with findings in western Ethiopia and to that of southwestern Ethiopia that reported a significant association between knowledge of participants about malaria and early treatment-seeking behaviour for malaria [14, 15]. A study conducted in Nigeria revealed that knowledge of the causes of malaria had a significant influence on health-seeking behaviour [8]. This is similar to the finding of a study in Burkina Faso [16]. Regarding participants’ experience of previous malaria illness in this study, those who had history of malaria visited health facilities earlier than those who had no history. This may be due to the fact that previous malaria history might have helped them to know malarial symptoms and seek treatment early. This study also showed that respondents who were less than 6 km from a health centre were more likely to seek treatment for malaria early than those who were 6 km or more from the same health centre. This is similar to previous findings in southern Ethiopia where patients from distant areas delayed health facility visits [15]. This finding is also slightly similar to a report in southwestern Ethiopia where patients who were 3 km or more from a health centre were more likely to delay treatment for malaria compared to those who were within 3 km [17]. This is consistent with a finding in Myanmar that reported a strong association between a 3-km distance and early attendance at health facilities following illness [6]. A study in Boudh district, India, reported that patients visited a health facility earlier when they were within 5 km than those who were outside this range [18]. A study in Guinea revealed that children who lived more than 3 km from a health facility were more likely to delay seeking care than those who lived closer [5]. It seems obvious that distance is an important predictor of early treatment-seeking for malaria.

## Conclusion

A low proportion of malaria-suspected patients sought treatment within 24 h of fever onset compared to the national target. This reduces the effectiveness of malaria control and elimination efforts due to its direct impact on treatment. Knowledge of the advantages of sleeping under a bed net, knowledge of mosquito breeding sites in the prevention of malaria, overall knowledge of malaria, previous history of malaria illness, and residence distance from a health centre were contributing factors to early treatment-seeking behaviour for malaria. Early treatment-seeking behaviour is key in the prevention and control of malaria. Health messaging or SBCC (Social and Behavioural Change Communication) may be crucial in advancing knowledge to the community about the advantage of seeking early treatment. In this regard, the government of Ethiopia has been distributing long-lasting, insecticide-treated bed nets and other vector control mechanisms. However, it seems there is much to be done on the behaviour of communities, especially regarding seeking early treatment. Strengthening SBCC will have a valuable contribution in avoiding problems related to treatment-seeking behaviour.

## Abbreviations

EFMOH: Ethiopian Federal Ministry of Health
EFY: Ethiopian fiscal year
RDTs: rapid diagnostic tests
WHO: World Health Organization
